# Postoperative recurrence of pancreatic cancer controlled for 9 months solely by severe carbohydrate restriction: ketogenic diet

**DOI:** 10.1007/s12328-025-02221-z

**Published:** 2025-09-16

**Authors:** Takahiro Einama, Naoto Yonamine, Masaki Hatakeyama, Sho Ogata, Kazuki Kobayashi, Hanae Shinada, Takazumi Tsunenari, Yasuhiro Takihata, Mikiya Takao, Hideki Ueno, Yoji Kishi

**Affiliations:** 1 Department of Surgery, National Defense Medical College, 3-2 Namiki, Tokorozawa, Saitama 359-8513 Japan; 2 Nakara Clinic, Osaka city, Osaka 542-0081 Japan; 3 Department of Pathology and Laboratory Medicine, National Defense Medical College, Tokorozawa, Saitama 359-8513 Japan

**Keywords:** Carbohydrate restriction, Ketogenic diet, Pancreatic cancer

## Abstract

Carbohydrate restriction (ketogenic diet) is a cancer treatment that reduces energy production by oxidative phosphorylation in mitochondria of cancer cells and increases it through anaerobic glycolysis in cytoplasm. We report a patient in whom progression of pancreatic cancer recurrence was suppressed solely by a ketogenic diet for 9-month post-surgery. A 60-year-old female with a diagnosis of pancreatic cancer underwent pancreatoduodenectomy after 3 cycles of chemotherapy with gemcitabine plus nab-paclitaxel. Multiple pulmonary metastases were observed 22 months after the surgery. We administered gemcitabine plus nab-paclitaxel for 1 year. As the partial response continued for 1 year, we performed radiotherapy for the remnant pulmonary metastases followed by administration of S-1 for 6 months. Ten months after radiotherapy, CT showed exacerbation of the pulmonary metastases. As treatment, she requested severe carbohydrate restriction. After 9 months of the ketogenic diet, CT revealed stable disease. A ketogenic diet may have the therapeutic effect of suppressing tumor progression if strictly applied.

## Introduction

The prognosis of patients with pancreatic ductal adenocarcinoma (PDAC) is generally poor, even in surgical cases. The postoperative recurrence rate exceeds 70% [1]. Systemic chemotherapy is the first choice of the treatment strategy for postoperative recurrence. However, the average survival period after recurrence is 6.8 to 11.1 months [2, 3].

Cancer cells exhibit decreases in energy production through oxidative phosphorylation in mitochondria but increases through anaerobic glycolysis in cytoplasm [4]. Warburg was the first to report enhanced, accelerated conversion of glucose to lactate in malignant tumors, even in the presence of abundant oxygen [5]. ^18^F-fluorodeoxyglucose positron emission tomography/computed tomography fusion imaging (FDG–PET) utilizes the property of cancer cells taking up large amounts of glucose to localize cancer [6, 7].

Carbohydrate restriction (ketogenic diet) is a dietary therapy that has been used primarily in the field of epilepsy. A ketogenic diet is still applied for drug-resistant epilepsy patients [8]. This type of diet is also a cancer treatment as it reduces energy production by oxidative phosphorylation in mitochondria of cancer cells and increases it through anaerobic glycolysis in cytoplasm, in other words, cancer cells obtain their energy mainly from glycolysis using glucose, and it may even be suitable for advanced cancer patients. It has no severe side effects and might improve aspects of quality of life and blood parameters in some patients with advanced metastatic tumors [9].

Herein, we report a patient in whom disease progression was suppressed for 9 months after postoperative recurrence of PDAC solely with a ketogenic diet.

## Case report

A 60-year-old female diagnosed with borderline resectable PDAC (PDAC contact with superior mesenteric artery less than 180°) underwent pancreatoduodenectomy after 3 cycles of chemotherapy with gemcitabine plus nab-paclitaxel. The postoperative course was uneventful, and she was discharged on postoperative day 17. Pathological findings were compatible with the diagnosis of PDAC. The tumor size was 3.1 cm, and lymph node metastasis was positive in 5 of 28 dissected nodes. The pathologic stage according to 8th UICC was III (pT3 pN1b pM0).

**Fig. 1:**
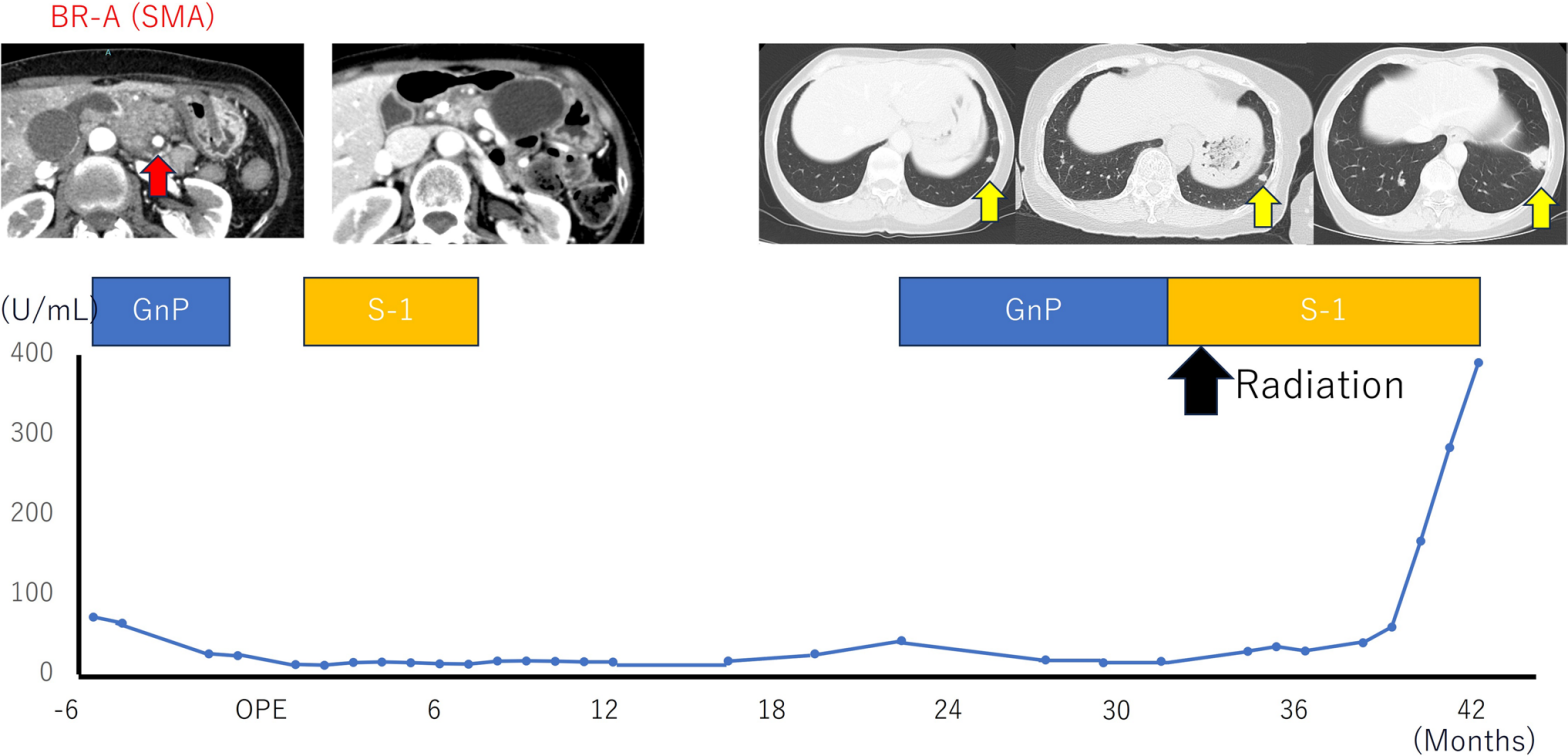
Changes in CA19-9 levels over the clinical course are summarized. Lung metastasis was noted 22 months after surgery. CT shows the increasing size of lung metastasis and CA19-9 levels after radiation. The red arrow indicates areas of pancreatic cancer. Yellow arrows show lung metastasis. *CA19-9* Carbohydrate antigen, *GnP* Gemcitabine plus nab-paclitaxel, *BR-A* Borderline resectable arterial invasion, *SMA* Superior mesenterial artery

She completed 6 months of postoperative adjuvant chemotherapy with S-1. Multiple pulmonary metastases were identified 22 months after surgery during outpatient follow-up. We administered gemcitabine plus nab-paclitaxel for 1 year. As her partial response continued for 1 year, we performed radiotherapy for the remnant pulmonary metastases followed by administration of S-1 for 6 months. Ten months after radiotherapy, CT showed progression of the pulmonary metastases (Fig. 1). We proposed re-administration of systemic chemotherapy, but she declined due to her previous adverse chemotherapy-related experience of impaired quality of life caused by peripheral neuropathy, fatigue and hair loss, so she requested a strict ketogenic diet instead.

### Ketogenic diet

**Fig. 2:**
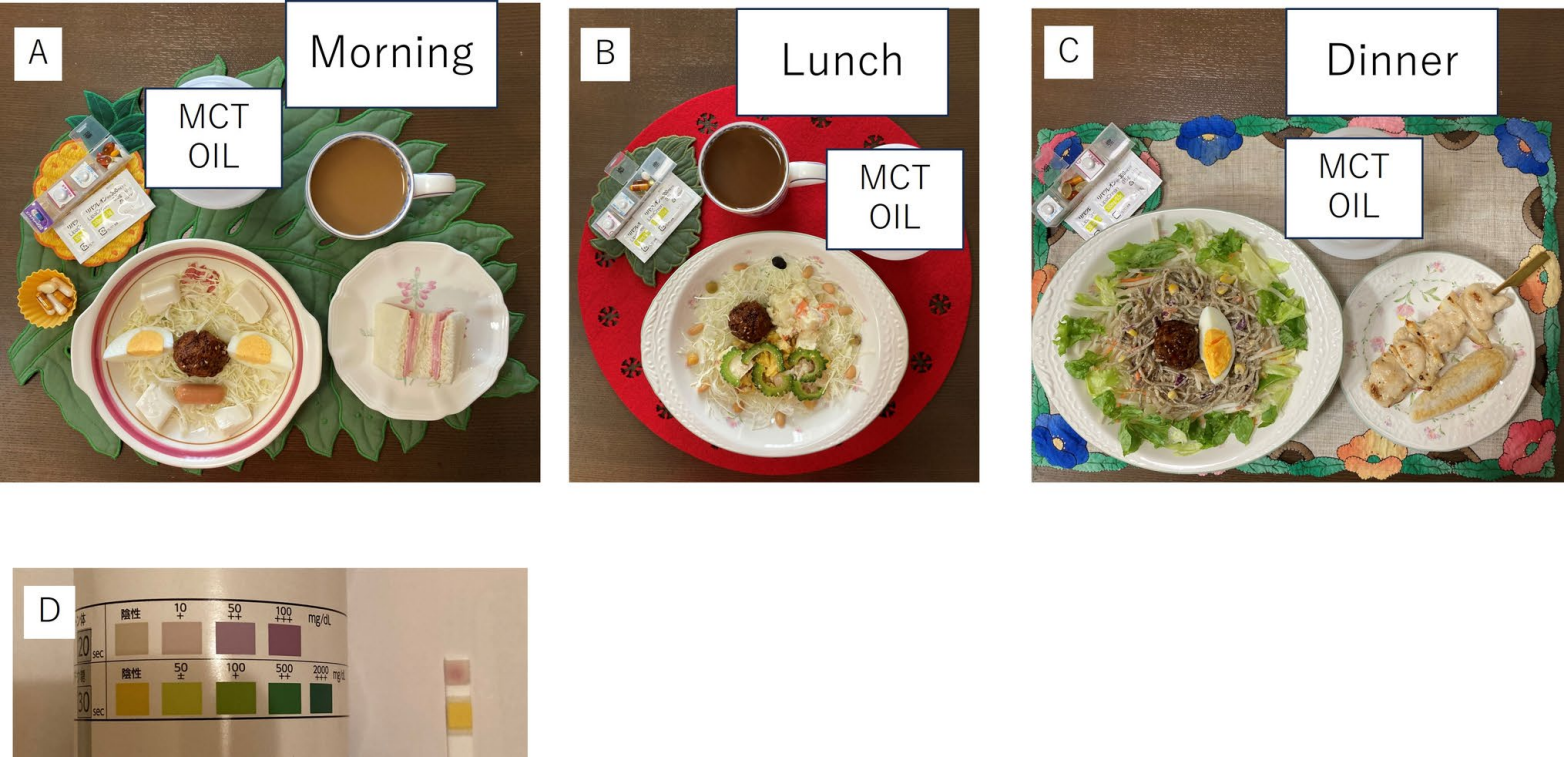
Samples of the ketogenic diet. **A** breakfast, carbohydrate: 14.1 g. **B** Lunch, carbohydrate: 13.6 g. **C** Dinner, carbohydrate: 13.8 g. **D** Patient checked her urine for ketones every morning using test strips. *MCT* medium chain triglyceride

The ketogenic diet comprised a total of about 1400 kcal per day. Carbohydrate intake was around 40–50 g/day (15 g/meal), protein intake was around 30–50 g/day, fat intake was around 85–105 g/day, and the amount of medium chain triglyceride oil was started from 10 g per meal, and gradually increased up to 20 g per meal [10]. When the patient follows a ketogenic diet, patient’s urine ketones will be positive because of the low carbohydrate content. Ketone bodies were checked using urine taken directly after waking up. First, the dietitian educated the patient on the amount of carbohydrates, fats, and proteins to be ingested in each meal. After that, the patient took a photo of each meal and sent it to the dietitian by email. The dietitian then looked at the photos and gave feedback every day for 3 months (Fig. 2).

### Clinical course during ketogenic diet

When she started the diet, her body weight, body fat percentage, and muscle mass were 43.3 kg, 30.4%, and 28.3 kg, respectively. After 4 months of the dietary therapy, she had lost 4.5 kg in body weight, but her muscle mass decrease was only 0.1 kg. In addition, her body fat had reduced by 3.6%. After starting the ketogenic diet, she reported that she did not feel hungry and felt great. She was especially pleased that she had no hair loss or peripheral neuropathy.

**Fig. 3:**
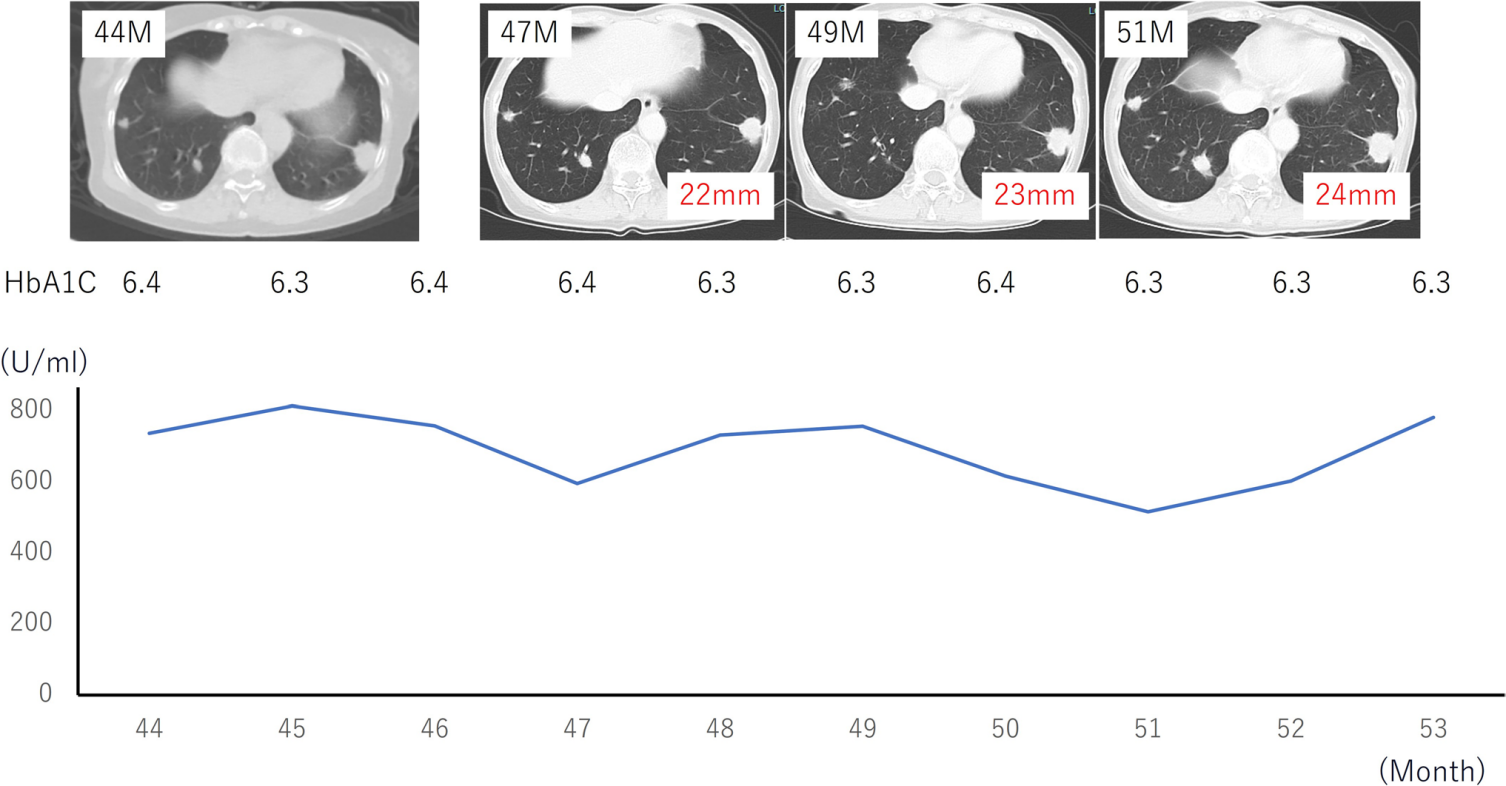
Clinical course after starting the ketogenic diet: after commencing the diet, there was no significant change in the tumor size regarding the lung metastasis, or CA19-9 and HbA1C level for 9 months

**Fig. 4:**
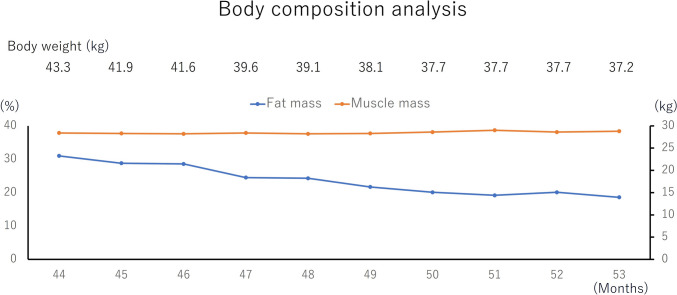
Body composition analysis: the muscle mass, amount of fat, amount of body fat, and body weight reduced, while the muscle mass was maintained

**Fig. 5:**
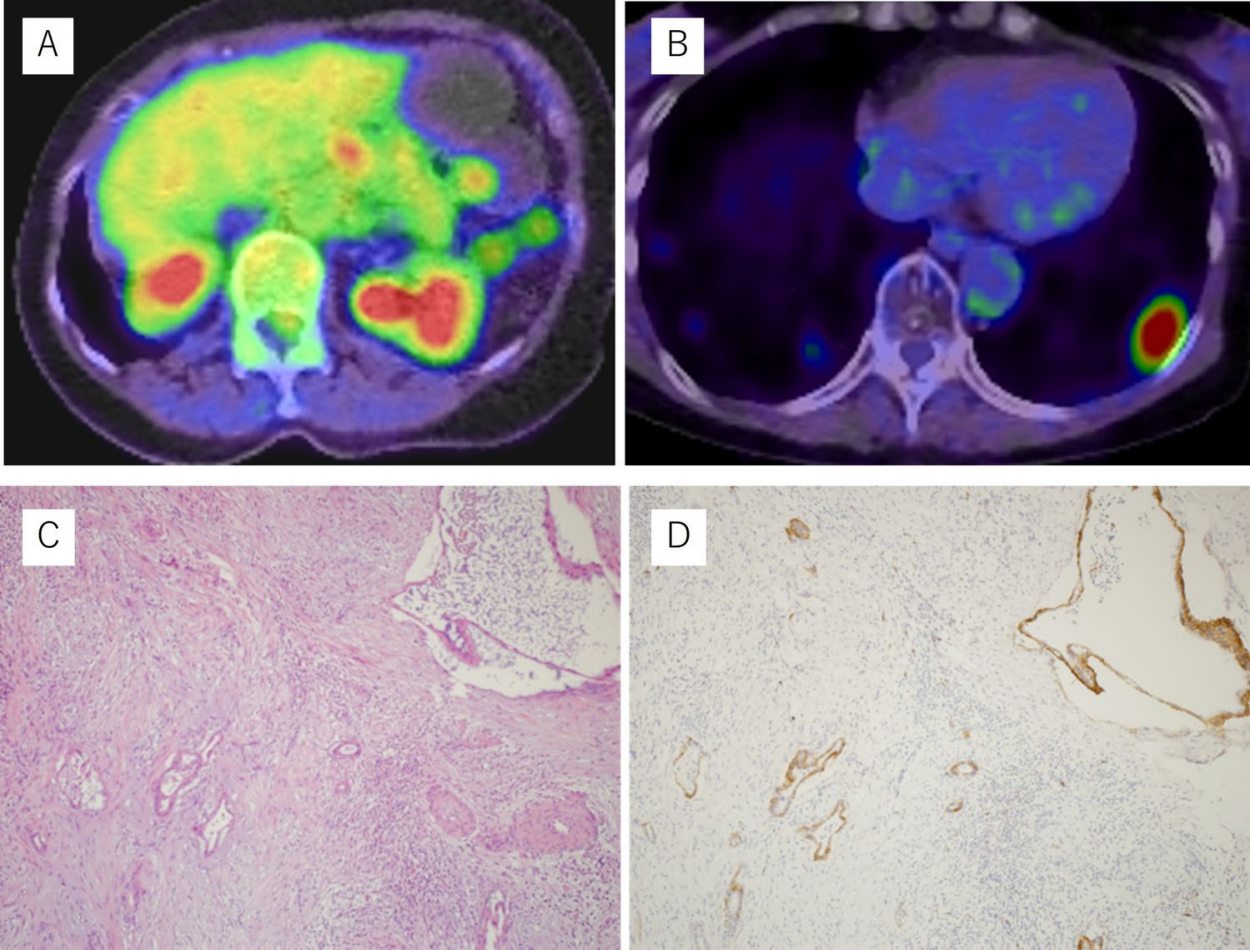
**A** Maximum intensity of the tumor in FDG–PET/CT, SUVmax was 5.7 at diagnosis. **B** SUVmax was 11.2 before ketogenic diet. **C** Cancer cells proliferate and infiltrate in irregular sized glands and small nests with marked desmoplastic stroma, typical features for pancreatic ductal adenocarcinoma. **D** Immunohistochemical stain for anti-glucose transporter-1 highlights cancer cells, reflecting their capacity of high glucose uptakes. We could find high expression anti-glucose transporter-1 in cancer cell. [Objective lens ×20; (**C**) hematoxylin and eosin, (**D**) diaminobenzidine]

After 9 months of the therapy, her body weight, body fat percentage, and muscle mass were 37.2 kg (−6.1 kg), 18.0% (−12.4%), and 28.7 kg (+0.4 kg), respectively (Fig. 3). In addition, after 9 months of the diet, serum CA19-9 remained almost unchanged, ranging from 738 to 783 U/mL. CT revealed stable disease, with a limited change in size from 23 to 24 mm (Fig. 4). In this case, up to 10 months after starting ketogenic diet, CT scan revealed stable disease. After 12 months of the dietary therapy, CA19-9 elevated 2142 U/mL and CT scan showed progressive disease of metastatic lung tumors. We added gemcitabine with continuing ketogenic diet for 7 months. In addition, we changed to Gemcitabine plus nab-paclitaxel for 2 months. Eventually, the coughing symptoms worsened and the patient was placed on best supportive care. The patient died 24 months after starting ketogenic diet. The patient’s tumor showed high expression of anti-glucose transporter-1, that reflects their capacity of high glucose uptakes. Therefore, it might be high possibility that ketogenic diet was effective (Fig. 5).

## Discussion

PDAC is associated with a poor prognosis if it recurs after curative surgery or is unresectable. Even with the use of anti-cancer drugs, it is difficult to achieve a survival period longer than 1 year [1–3]. However, in the present patient, the progression of a metastatic lung tumor was suppressed solely by the ketogenic diet. To the best of our knowledge, this is the first report of a patient with metastatic PDAC who achieved stable disease for 9 months solely through ketogenic diet therapy.

The ketogenic diet is a high-fat diet with limited carbohydrate intake, which uses fats as the main source of energy and is a therapeutic diet aimed at inducing ketosis. Tumor patients exhibit increased peripheral demands for fatty acids and protein. However, tumors utilize glucose as their main source of energy. Thus, a diet supplying the cancer patient with sufficient fats and proteins to meet demands while limiting carbohydrates to restrict energy supply could be a helpful strategy. A ketogenic diet fulfills these requirements [9]. When carbohydrates are deficient in the body due to ketogenic diet, fat is broken down and ketone bodies are produced. The patient checked her urine for ketones by herself every morning using test strips. In fact, the possibility of using it was examined in model mice with various types of cancer [11, 12]. Furthermore, the positive effect of the diet is also promoted by the ketone body *β*-hydroxybutyrate (BHB), which reduces the proliferation of colonic crypt cells and potently suppresses intestinal tumor growth [13].

Hagiwara et al. conducted a case–control study of ketogenic diet in patients with stage IV cancer. Carbohydrates were restricted to 10 g/day during week 1, 20 g/day from week 2 for 3 months, and 30 g/day thereafter. A total of 55 patients participated in the study, and data from 37 patients administered the ketogenic diet for 3 months were analyzed. After starting the ketogenic diet, patient’s serum BHB levels were significantly increased from 1 week to 3 months. The median overall survival was 32.2 (maximum 80.1) months, and the 3-year survival rate was 44.5%, and chemotherapy was administered to 89% of patients. These results demonstrated that the ketogenic diet promoted stable adherence and induced functional ketosis with high reproducibility, and was well-controlled in advanced cancer patients receiving chemotherapy. These results suggest that the ketogenic diet and chemotherapy have a synergistic effect in the treatment of cancer, as noted in mouse models [14].

The ketogenic diet has the advantage of not causing nausea, vomiting, hair loss, or bone marrow suppression, as can occur with anti-cancer drugs. Furthermore, although body fat is reduced, maintaining of skeletal muscle weight is observed. If there is a problem, there is a possibility that deficiencies in certain nutrients may develop: restricting grains might lead to vitamin B1, zinc, selenium, and magnesium deficiencies; limiting vegetables may result in calcium and iron deficiencies; restricting fruits might cause vitamin C deficiency. Thus, it may be important to perform regular evaluations such monitoring trace elements, and provide supplementation in cases of deficiency.

In conclusion, we encountered a PDAC patient who could achieve stable disease for 9 months solely with a ketogenic diet. Unlike chemotherapy, a ketogenic diet typically does not involve any risk of adverse cytotoxic events. Thus, it would be more tolerable and may be effective to suppress tumor progression if strictly applied.

## References

[1] Einama T, Takihata Y, Aosasa S, et al. Prognosis of pancreatic cancer based on resectability: a single center experience. Cancers (Basel). 2023. 10.3390/cancers15041101. 36831444 10.3390/cancers15041101 PMC9954753

[2] Conroy T, Desseigne F, Ychou M, et al. Folfirinox versus gemcitabine for metastatic pancreatic cancer. N Engl J Med. 2011;364:1817–25. 21561347 10.1056/NEJMoa1011923

[3] Von Hoff DD, Ervin T, Arena FP, et al. Increased survival in pancreatic cancer with nab-paclitaxel plus gemcitabine. N Engl J Med. 2013;369:1691–703. 24131140 10.1056/NEJMoa1304369 PMC4631139

[4] Vaupel P, Multhoff G. Revisiting the Warburg effect: historical dogma versus current understanding. J Physiol. 2021;599:1745–57. 33347611 10.1113/JP278810

[5] Hardie DG. 100 years of the Warburg effect: a historical perspective. Endocr Relat Cancer. 2022;29:T1–13. 36094878 10.1530/ERC-22-0173

[6] Lammertsma AA. Forward to the past: the case for quantitative PET imaging. J Nucl Med. 2017;58:1019–24. 28522743 10.2967/jnumed.116.188029

[7] Einama T, Yamagishi Y, Takihata Y, et al (2022) Clinical impact of dual time point ^18^F-Fluorodeoxyglucose positron emission tomography/computed tomography fusion imaging in pancreatic cancer. Cancers (Basel). 10.3390/cancers14153688. 10.3390/cancers14153688 PMC9367454 35954351

[8] Kwan P, Schachter SC, Brodie MJ. Drug-resistant epilepsy. N Engl J Med. 2011;365:919–26. 21899452 10.1056/NEJMra1004418

[9] Schmidt M, Pfetzer N, Schwab M, et al. Effects of a ketogenic diet on the quality of life in 16 patients with advanced cancer: a pilot trial. Nutr Metab (Lond). 2011;8:54. 21794124 10.1186/1743-7075-8-54 PMC3157418

[10] Fujii T, Ito Y, Takahashi S, et al. Outcome of ketogenic diets in GLUT1 deficiency syndrome in Japan: a nationwide survey. Brain Dev. 2016;38:628–37. 26923720 10.1016/j.braindev.2016.01.002

[11] Allen BG, Bhatia SK, Buatti JM, et al. Ketogenic diets enhance oxidative stress and radio-chemo-therapy responses in lung cancer xenografts. Clin Cancer Res. 2013;19:3905–13. 23743570 10.1158/1078-0432.CCR-12-0287 PMC3954599

[12] Abdelwahab MG, Fenton KE, Preul MC, et al. The ketogenic diet is an effective adjuvant to radiation therapy for the treatment of malignant glioma. PLoS ONE. 2012;7:e36197. 22563484 10.1371/journal.pone.0036197 PMC3341352

[13] Dmitrieva-Posocco O, Wong AC, Lundgren P, et al. Beta-Hydroxybutyrate suppresses colorectal cancer. Nature. 2022;605:160–5. 35477756 10.1038/s41586-022-04649-6 PMC9448510

[14] Hagihara K, Kajimoto K, Osaga S, et al (2020) Promising effect of a new ketogenic diet regimen in patients with advanced cancer. Nutrients 12(5):1473. 10.3390/nu12051473 PMC7284721 32438645

